# Character

**Published:** 1906-10

**Authors:** Gustavus North

**Affiliations:** Cedar Rapids, Iowa.


### CHARACTER.

By Dr. Gustavus North, Cedar Rapids, Iowa.

Life is the expression of force, the period between birth and death. It should be our highest aim to make the most of this life; to live soberly, righteously and unselfishly in every motive.

In teaching it has always been my aim to impress upon the minds of young men the importance of character; to seek always that which is conducive to spiritual and mental growth, and to shun all that has a tendency to draw one from the highest and noblest thoughts of life.

Young men, did you ever stop to think during life's journey how little it takes to mar the character of an individual? A person's character is formed similar to the formation of an icicle, which commences to form from a single drop of water; drop after drop forms a clear transparent crystal; when by some mishap a drop of clouded water appears and is very distinctly seen mingled with the crystals, then again the pure drops follow, and a great crystal is formed, but still the clouded drop remains mingled, and inseparable in form, only makes the clouded streaks more conspicuous. Just so in regard to life—one misstep, one unmanly act, will cloud your character through life and leave an imprint on life that cannot be erased.

Did you ever take an object at some distant point and see how straight you could walk to that point, not a crook or turn in the way? Again, have you not tried the same thing, and stopped on the way perhaps for some trifling thing, and when you would continue your march you would have a crook in your pathway?

How often we look back on our lives and see the little crooks and clouded drops that we would give our lives to remove, but they are there stamped upon our character. Each individual is engraving his own monument. Let each one live a life worthy of praise, and our monument will be unveiled with a clear inscription of the power we have maintained.

